# Tirzepatide versus other glucose-lowering therapies and risk of COPD exacerbations in patients with comorbid type 2 diabetes: a retrospective cohort study

**DOI:** 10.3389/fphar.2026.1917340

**Published:** 2026-10-09

**Authors:** Wan-Hsuan Hsu, Yang-En Chan, Jheng-Yan Wu, Yao-Ming Chuang, Chia-Yu Kuo, Bo-Wen Shiau, Po-Yu Huang, Chih-Cheng Lai

**Affiliations:** 1 Department of Internal Medicine, Chi Mei Medical Center, Tainan, Taiwan; 2 Department of General Medicine, Chi Mei Medical Center, Tainan, Taiwan; 3 Department of Nutrition, Chi Mei Medical Center, Tainan, Taiwan; 4 Department of Public Health, College of Medicine, National Cheng Kung University, Tainan, Taiwan; 5 Department of Intensive Care Medicine, Chi Mei Medical Center, Tainan, Taiwan; 6 School of Medicine, College of Medicine, National Sun Yat-sen University, Kaohsiung, Taiwan

**Keywords:** chronic obstructive pulmonary disease, COPD exacerbation, incretin-based therapy, mortality, tirzepatide, type 2 diabetes

## Abstract

**Background:**

Chronic obstructive pulmonary disease (COPD) and type 2 diabetes (T2D) frequently coexist, sharing inflammatory and metabolic pathways. Tirzepatide (TZP), a dual glucose-dependent insulinotropic polypeptide and glucagon-like peptide-1 receptor agonist, has demonstrated metabolic and anti-inflammatory effects. However, its impact on pulmonary outcomes in patients with COPD and T2D remains uncertain.

**Methods:**

Using the TriNetX global research network, we conducted a retrospective cohort study of adults with coexisting COPD and T2D prescribed TZP, sodium-glucose cotransporter-2 inhibitors (SGLT2i), dipeptidyl peptidase-4 inhibitors (DPP-4i), or glucagon-like peptide-1 receptor agonists (GLP-1RA). Propensity score matching (1:1) was performed separately for each comparison. Primary outcome was COPD exacerbation; secondary outcomes included all-cause mortality, acute respiratory failure, and lower respiratory tract infection. Outcomes were assessed during a prespecified observation period from day one through day 365 after the index date.

**Results:**

Matched populations included 6,080 patients per group (TZP–SGLT2i), 4,684 (TZP–DPP-4i), and 6,727 (TZP–GLP-1RA). In the independently matched comparisons, median follow-up was 232 versus 365 days for TZP versus SGLT2i, 235 versus 365 days for TZP versus DPP-4i, and 233 versus 365 days for TZP versus other GLP-1RAs; incidence rates were expressed per 1,000 person-years. Compared with SGLT2i and DPP-4i, TZP significantly reduced COPD exacerbation risk (hazard ratio [HR] 0.73 [95% CI 0.64–0.82] and 0.72 [95% CI 0.63–0.82], respectively) and all-cause mortality (HR 0.30 [95% CI 0.23–0.38] and 0.24 [95% CI 0.18–0.31], respectively). TZP also reduced acute respiratory failure and lower respiratory tract infection risks. Outcomes were similar between TZP and GLP-1RA, except TZP showed lower mortality (HR 0.55 [95% CI 0.42–0.72]).

**Conclusion:**

Among patients with COPD and T2D, TZP use was associated with lower risks of COPD exacerbations, respiratory complications, and mortality compared with SGLT2i and DPP-4i, findings that warrant confirmation through randomized clinical trials.

## Introduction

Chronic obstructive pulmonary disease (COPD) and type 2 diabetes (T2D) frequently coexist, reflecting shared risk factors such as aging, smoking, systemic inflammation, and obesity (Hughes et al., 2020; de Oca et al., 2025). Epidemiologic studies suggest that individuals with T2D have a higher prevalence of COPD and experience more frequent exacerbations, hospitalizations, and mortality compared with those without diabetes (Anghel et al., 2025). Conversely, COPD-related hypoxia, corticosteroid use, and systemic inflammation may exacerbate insulin resistance and glycemic dysregulation, creating a bidirectional pathophysiologic interplay (de Oca et al., 2025). This complex interaction is further compounded by overlapping comorbidities, including cardiovascular disease and metabolic syndrome, which contribute to poor clinical outcomes (Hughes et al., 2020; Ji et al., 2025; Gläser et al., 2015; Müllerova et al., 2013). Despite the high burden of coexisting COPD and T2D, therapeutic strategies that address both conditions remain limited (de Oca et al., 2025). Current antidiabetic agents vary in their effects on pulmonary outcomes, and few have been systematically evaluated for their impact on COPD exacerbations (Chang et al., 2025; Meng et al., 2025; Pirera et al., 2025; Ray et al., 2025). Identifying agents that offer dual benefit for metabolic and respiratory health is therefore a critical unmet need in this high-risk population.

Glucagon-like peptide-1 receptor agonists (GLP-1RAs) have been the most extensively studied incretin-based therapy in this context. Several observational and pharmacoepidemiologic studies have reported that GLP-1RA use is associated with reduced COPD exacerbations, respiratory hospitalizations, and mortality compared with other glucose-lowering agents (Foer et al., 2023; Huang et al., 2026; Yen et al., 2024). However, these studies vary in design, comparator selection, and outcome ascertainment, and most rely on claims-based or single-center cohorts with limited ability to fully address confounding by indication, obesity, and disease severity (Foer et al., 2023; Huang et al., 2026; Yen et al., 2024). Taken together, this body of clinical evidence supports a possible respiratory effect of GLP-1RA therapy, but comparative data across incretin-based agents, including newer dual agonists, remain sparse.

Tirzepatide (TZP) is a novel dual glucose-dependent insulinotropic polypeptide (GIP) and glucagon-like peptide-1 (GLP-1) receptor agonist that has demonstrated superior efficacy in glycemic control and weight reduction compared with other antidiabetic therapies (Aronne et al., 2025; Frías et al., 2021; Jastreboff et al., 2025). The biological rationale for a pulmonary benefit of TZP is derived largely from preclinical and mechanistic work rather than clinical studies in patients with COPD. Animal and *in vitro* studies suggest that dual GIP and GLP-1 receptor activation can attenuate airway inflammation, reduce oxidative stress, and modulate immune responses implicated in chronic lung disease (Janić et al., 2024). Similarly, evidence for anti-inflammatory and endothelial-protective effects of GLP-1 receptor agonism is drawn substantially from asthma models and mechanistic studies rather than COPD-specific clinical cohorts (Foer et al., 2023; Sun et al., 2017; Wang et al., 2023). Clinically, TZP’s effects on weight loss and insulin sensitivity in patients with T2D may plausibly improve pulmonary mechanics and reduce the burden of comorbid conditions (Corrao et al., 2024), but whether this translates into a measurable reduction in COPD exacerbations has not been directly tested.

Given the existing clinical evidence supporting a respiratory effect of GLP-1RA in patients with COPD, the key unanswered question is whether TZP, through its additional GIP-receptor activity and greater metabolic efficacy, confers further benefit beyond that already observed with GLP-1RA, rather than whether incretin-based therapy has any pulmonary effect at all. To address this gap, we conducted a retrospective cohort study using the TriNetX global research network to evaluate the comparative effectiveness of TZP versus other commonly prescribed glucose-lowering agents in reducing the risk of COPD exacerbations and all-cause mortality among patients with coexisting COPD and T2D. This analysis aimed to determine whether TZP is associated with respiratory outcomes that are distinguishable from those of existing GLP-1RA therapy in real-world clinical practice.

## Methods

### Data source

We conducted this retrospective cohort study using the TriNetX global federated health research network, a platform that aggregates de-identified electronic health records from healthcare organizations (HCOs) across multiple countries (Wilson et al., 2024). The TriNetX platform provides access to longitudinal data, including demographics, diagnoses, procedures, medications, laboratory values, and clinical outcomes. All data were de-identified in compliance with the Health Insurance Portability and Accountability Act (HIPAA) and the platform has received a waiver from the Western Institutional Review Board. This study adhered to the Strengthening the Reporting of Observational Studies in Epidemiology (STROBE) reporting guideline.

### Study population

Within the TriNetX network, individuals who had accessed healthcare services at least three times between 1 June 2022, and 30 May 2025. From this population, patients with coexisting diagnoses of COPD and T2D were identified using International Classification of Diseases, 10th Revision, Clinical Modification (ICD-10-CM) codes. COPD was identified using codes J41–44, and T2D was identified using codes E11.x (Hsu et al., 2025).

Inclusion criteria were: (1) diagnosis of both COPD and T2D; (2) age 40 years or older at index date since COPD is uncommon before this age; and (3) initiation of TZP, SGLT2i, DPP-4i, and GLP-1RA therapy between 1 June 2022, and 30 May 2025, which was chosen because TZP was approved in 2022. The index date was defined as the date of the first prescription for the study medication.

Exclusion criteria were: (1) prior use of the study medication or comparator medication within 1 year before the index date, and (2) diagnosis of type 1 diabetes, gestational diabetes, or other specific types of diabetes.

### Study design and exposure definition

We employed an active-comparator, new-user design (Yoshida et al., 2015) to minimize confounding by indication and ensure balanced comparison groups. Three separate cohorts were constructed: (1) TZP users versus SGLT2i users, (2) TZP users versus DPP-4i users, and (3) TZP users versus GLP-1RA users. The SGLT2i group included patients prescribed bexagliflozin, canagliflozin, dapagliflozin, empagliflozin, ertugliflozin, or sotagliflozin. The DPP-4i group included patients prescribed alogliptin, linagliptin, sitagliptin, saxagliptin, or vildagliptin. The GLP-1RA group included patients prescribed any GLP-1 RA agent, including albiglutide, dulaglutide, exenatide, lixisenatide, liraglutide, and semaglutide.

Cohort entry (time zero) was defined as the date of the first prescription for the respective exposure drug, and follow-up began immediately from this index date, so that no patient contributed follow-up time prior to meeting exposure criteria. This design inherently avoids immortal time bias, as treatment status and the start of follow-up are aligned at the same index date for all patients, with no exposure-free grace period or delayed entry window during which outcome events could not be observed. Patients were assigned to their initial exposure group based on the first qualifying prescription and were followed under an intention-to-treat framework, regardless of subsequent treatment discontinuation, switching, or dose adjustment; treatment persistence and adherence after cohort entry were not assessed.

Follow-up duration was summarized using the median and interquartile range. Total person-years were calculated by multiplying the number of patients in each cohort by the corresponding unrounded mean follow-up duration and dividing by 365. The specific codes used for cohort identification are listed in Supplementary Table S1 and the temporal relationship among the pre-index washout period, index date, and outcome observation period is illustrated in Supplementary Figure S1.

### Propensity score matching

To balance baseline characteristics between treatment groups and reduce confounding, 1:1 propensity score matching (PSM) was performed using a greedy nearest-neighbor algorithm with a caliper width of 0.1 standard deviations of the logit of the propensity score. Covariate balance after matching was assessed using standardized mean differences, with values < 0.1 considered indicative of adequate balance. Propensity scores were estimated using multivariable logistic regression models that included the following covariates: age, sex, race, ethnicity, body mass index (BMI), hemoglobin A1c (HbA1c) level, smoking status, baseline comorbidities, and baseline glucose-lowering agents. Covariates were selected *a priori* based on established clinical relevance to both treatment choice and study outcomes, consistent with prior pharmacoepidemiologic studies using this exposure comparison; no interaction terms were included in the propensity score model. As the TriNetX platform performs matching using its built-in analytics tool on the basis of available structured data, patients with missing covariate values were handled according to the platform’s default approach, which excludes patients with incomplete data on the specified matching variables rather than imputing missing values. Balance between groups after PSM was assessed using standardized mean differences (SMDs), with an SMD of less than 0.10 indicating adequate balance. The details of the matching process are provided in Supplementary Table S2.

### Outcomes

The primary outcome was COPD exacerbation. Secondary outcomes included: (1) all-cause mortality; (2) acute respiratory failure; and (3) lower respiratory tract infection. Patients were followed from the index date for 1 year, or until the occurrence of an outcome event, death, or loss to follow-up, whichever occurred first. The identification of these outcomes was based on diagnostic and procedural codes, which are delineated in Supplementary Table S3.

### Negative outcomes

To evaluate potential residual confounding and assess the specificity of the observed associations, two negative control outcomes, including skin cancer and cataract, were prespecified. These outcomes were selected because they are biologically implausible as direct consequences of TZP and other glucose-lowering therapies. Outcome definitions and corresponding diagnostic codes are provided in Supplementary Table S3. Negative outcomes were analyzed using the same cohort definitions, matching procedures, follow-up, and statistical models as those applied in the primary and secondary outcome analyses.

### Statistical analysis

Baseline characteristics were summarized using frequencies and percentages for categorical variables and means with standard deviations or medians with interquartile ranges for continuous variables, as appropriate. We compared characteristics between treatment groups using SMDs before and after PSM.

Incidence rates for outcomes were calculated as the number of events per 1,000 person-years. We used Cox proportional hazards regression models to estimate hazard ratios (HRs) and 95% confidence intervals (CIs) for the association between TZP use and each outcome compared with SGLT2i, DPP-4i or GLP-1RA use. The analyses were performed using the built-in TriNetX analytic function. Because matched-pair identifiers were not available, pair-clustered robust standard errors could not be calculated, and the matching was not explicitly accounted for in the standard error estimation. The proportional hazards assumption was assessed using Schoenfeld residuals. Kaplan-Meier curves were generated to visualize cumulative incidence of the primary outcome, and differences between groups were assessed using the log-rank test.

To assess the robustness of our findings to unmeasured confounding, we calculated E-values, defined as the minimum strength of association that an unmeasured confounder would need to have with both the exposure and outcome to fully explain away the observed association (Haneuse et al., 2019).

We performed prespecified stratified analyses to evaluate consistency of treatment associations across clinically relevant subgroups defined by age (40–64 years vs. ≥ 65 years), sex (female vs. male), recent history of COPD exacerbation (yes vs. no), HbA1c level (<9% vs. ≥ 9%), and BMI (<25 kg/m^2^ vs. ≥ 25 kg/m^2^). The codes used to identify these subgroups are displayed in Supplementary Table S4. For each subgroup analysis, PSM was performed *de novo* within the respective subgroup to ensure optimal balance of baseline characteristics. Interactions between treatment and subgroup variables were assessed using likelihood ratio tests. Given the number of subgroup comparisons, all subgroup analyses were considered exploratory.

## Results

### Patients’ selection

Data were obtained from the TriNetX global Collaborative Network, which included 195,140,211 individuals as of 12 October 2025. From this database, 1,551,038 patients aged above 40 years with COPD and T2D who had at least three healthcare encounters between 1 June 2022, and 30 May 2025, were identified. After applying study exclusion criteria, three comparison cohorts were established according to exposure to TZP versus SGLT2i, DPP-4i, or GLP-1RA. To reduce potential confounding, 1:1 PSM was performed separately for each comparison. The final matched populations included 6,080 per group in the TZP–SGLT2i cohort, 4,684 patients per group in the TZP–DPP-4i cohort, and 6,727 per group in the TZP–GLP-1RA cohort (Figure 1). During the prespecified observation period from day 1 through day 365 after the index date, median follow-up was 232 versus 365 days, 235 versus 365 days, and 233 versus 365 days in the TZP versus SGLT2i, DPP-4i, and other GLP-1RA comparisons, respectively. Detailed follow-up distributions, including the interquartile ranges and total person-years, are provided in Supplementary Table S5.

**FIGURE 1:**
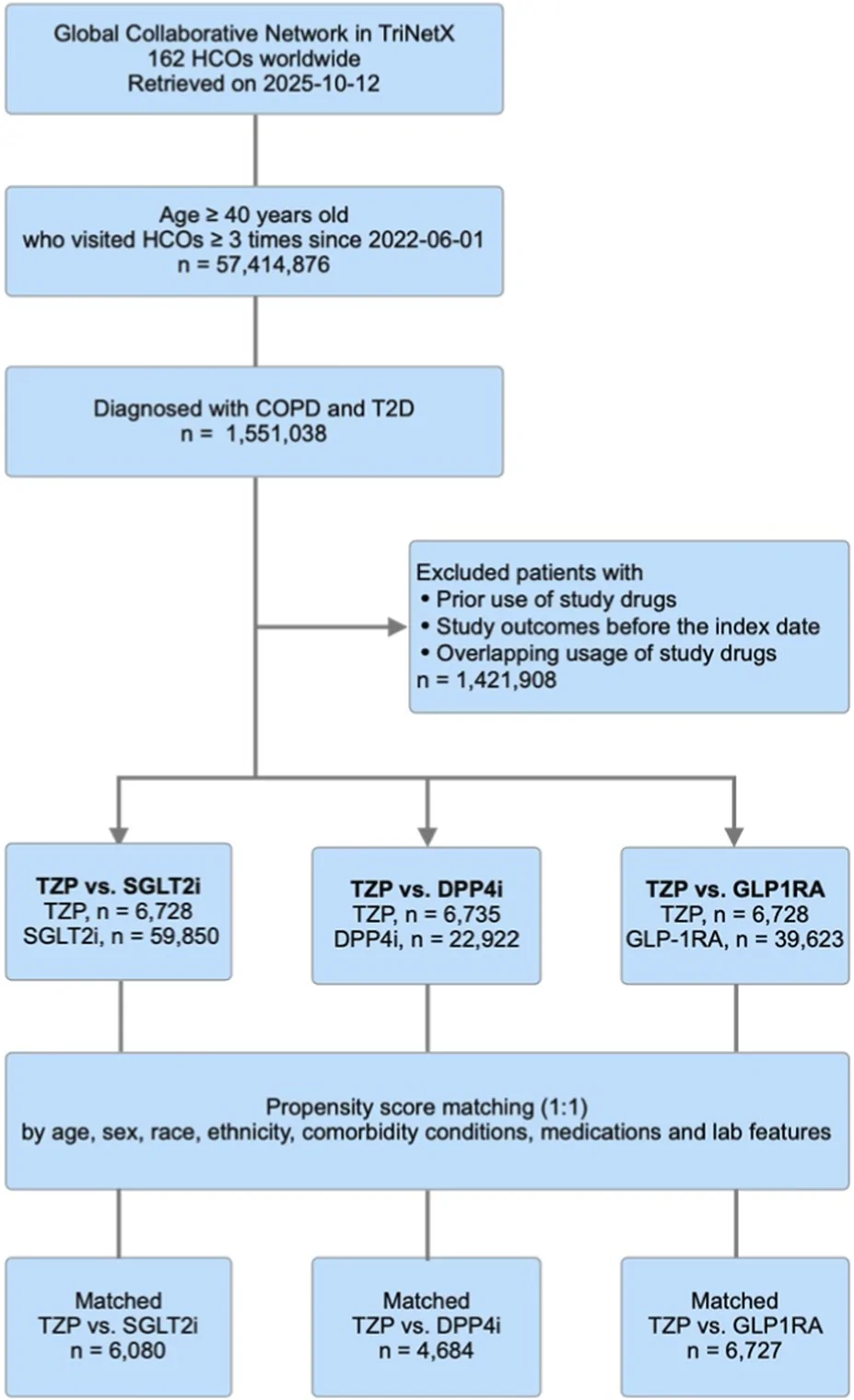
Flowchart of cohort construction. DPP-4i, dipeptidyl peptidase four inhibitor; GLP-1RA, glucagon-like peptide-1 receptor agonists; HCO, healthcare organizations; TZP, tirzepatide; SGLT2i, sodium-glucose cotransporter two inhibitor.

### Baseline characteristics

Before matching, substantial baseline differences were observed between patients treated with TZP and those receiving SGLT2i, DPP-4i, or GLP-1RA (Supplementary Table S6). Following PSM, demographic and clinical variables, including age, sex, race, BMI, HbA1c, comorbidities, T2D-related complications, and concomitant use of cardiovascular, antihypertensive, and glucose-lowering agents, were well-balanced across all matched cohorts (TZP vs. SGLT2i, TZP vs. DPP-4i, and TZP vs. GLP-1RA). All SMDs were below 0.1, confirming excellent covariate balance (Table 1).

**TABLE 1:** Post-matching baseline characteristics of the tirzepatide (TZP) cohort and comparator cohorts.

Comparator therapy	TZP group (n = 6,080)	SGLT2i group (n = 6,080)	SMD	TZP group (n = 4,684)	DPP-4i group (n = 4,684)	SMD	TZP group (n = 6,727)	GLP-1RA group (n = 6,727)	SMD
Demographics									
Age at index (mean ± SD)	65.1 ± 9.2	65.0 ± 10.0	0.011	66.7 ± 8.7	66.4 ± 10.1	0.031	64.3 ± 9.6	64.4 ± 9.8	0.011
Sex									
Female	3576 (58.8)	3584 (58.9)	0.003	2729 (58.3)	2745 (58.6)	0.007	4131 (61.4)	4162 (61.9)	0.009
Male	2500 (41.1)	2495 (41.0)	0.002	1954 (41.7)	1936 (41.3)	0.008	2593 (38.5)	2561 (38.1)	0.010
Race									
White	4704 (77.4)	4759 (78.3)	0.022	3540 (75.6)	3522 (75.2)	0.009	5262 (78.2)	5279 (78.5)	0.006
Black or African. American	892 (14.7)	863 (14.2)	0.014	720 (15.4)	761 (16.2)	0.024	957 (14.2)	946 (14.1)	0.005
Asian	69 (1.1)	55 (0.9)	0.023	65 (1.4)	66 (1.4)	0.002	70 (1.0)	51 (0.8)	0.030
American Indian or Alaska Native	41 (0.7)	44 (0.7)	0.006	32 (0.7)	35 (0.7)	0.008	44 (0.7)	40 (0.6)	0.008
Native Hawaiian or other Pacific. Islander	39 (0.6)	45 (0.7)	0.012	29 (0.6)	33 (0.7)	0.011	42 (0.6)	51 (0.8)	0.016
Other race	170 (2.8)	169 (2.8)	0.001	136 (2.9)	129 (2.8)	0.009	185 (2.8)	172 (2.6)	0.012
Unknown race	165 (2.7)	145 (2.4)	0.021	162 (3.5)	138 (2.9)	0.029	167 (2.5)	188 (2.8)	0.019
Ethnicity									
Hispanic or Latino	288 (4.7)	272 (4.5)	0.013	219 (4.7)	230 (4.9)	0.011	313 (4.7)	302 (4.5)	0.008
Not Hispanic or Latino	4551 (74.9)	4574 (75.2)	0.009	3459 (73.8)	3547 (75.7)	0.043	5054 (75.1)	5149 (76.5)	0.033
Unknown ethnicity	1241 (20.4)	1234 (20.3)	0.003	1006 (21.5)	907 (19.4)	0.052	1360 (20.2)	1276 (19)	0.031
Lifestyle									
Nicotine dependence	1293 (21.3)	1282 (21.1)	0.004	980 (20.9)	1003 (21.4)	0.012	1366 (20.3)	1318 (19.6)	0.018
Personal history of nicotine dependence	1733 (28.5)	1728 (28.4)	0.002	1284 (27.4)	1279 (27.3)	0.002	1913 (28.4)	1881 (28)	0.011
Problem with socioeconomic circumstances	283 (4.7)	281 (4.6)	0.002	225 (4.8)	238 (5.1)	0.013	317 (4.7)	301 (4.5)	0.011
Comorbid conditions									
COPD exacerbation	708 (11.6)	713 (11.7)	0.003	560 (12.0)	572 (12.2)	0.008	744 (11.1)	754 (11.2)	0.005
Metabolic disorders									
Diabetes mellitus	4449 (73.2)	4550 (74.8)	0.038	3553 (75.9)	3613 (77.1)	0.030	4813 (71.5)	4794 (71.3)	0.006
Overweight and obesity	2908 (47.8)	2929 (48.2)	0.007	1939 (41.4)	1966 (42.0)	0.012	3481 (51.7)	3425 (50.9)	0.017
Cardiovascular diseases									
Hypertensive diseases	4323 (71.1)	4402 (72.4)	0.029	3285 (70.1)	3311 (70.7)	0.012	4766 (70.8)	4753 (70.7)	0.004
Ischemic heart diseases	1774 (29.2)	1752 (28.8)	0.008	1360 (29.0)	1381 (29.5)	0.010	1819 (27.0)	1813 (27)	0.002
Cerebrovascular diseases	520 (8.6)	508 (8.4)	0.007	455 (9.7)	475 (10.1)	0.014	543 (8.1)	546 (8.1)	0.002
Pulmonary heart disease	538 (8.8)	517 (8.5)	0.012	400 (8.5)	423 (9.0)	0.017	572 (8.5)	505 (7.5)	0.037
Heart failure	1131 (18.6)	1161 (19.1)	0.013	888 (19)	930 (19.9)	0.023	1135 (16.9)	1115 (16.6)	0.008
Chronic kidney diseases									
Stage 3	758 (12.5)	813 (13.4)	0.027	632 (13.5)	678 (14.5)	0.028	777 (11.6)	772 (11.5)	0.002
Stage 4	136 (2.2)	129 (2.1)	0.008	129 (2.8)	151 (3.2)	0.028	139 (2.1)	159 (2.4)	0.020
End stage renal disease	139 (2.3)	160 (2.6)	0.022	144 (3.1)	156 (3.3)	0.015	159 (2.4)	166 (2.5)	0.007
Others									
Schizophrenia	96 (1.6)	99 (1.6)	0.004	84 (1.8)	93 (2)	0.014	108 (1.6)	90 (1.3)	0.022
Mood disorders	1699 (27.9)	1697 (27.9)	0.001	1203 (25.7)	1240 (26.5)	0.018	1981 (29.4)	1993 (29.6)	0.004
Lung cancer	92 (1.5)	96 (1.6)	0.005	81 (1.7)	81 (1.7)	<0.001	95 (1.4)	105 (1.6)	0.012
Liver cirrhosis	165 (2.7)	162 (2.7)	0.003	136 (2.9)	134 (2.9)	0.003	180 (2.7)	181 (2.7)	0.001
Obstructive sleep apnea	2040 (33.6)	2063 (33.9)	0.008	1278 (27.3)	1277 (27.3)	<0.001	2422 (36)	2332 (34.7)	0.028
Medications									
Biguanides	1727 (28.4)	1776 (29.2)	0.018	1296 (27.7)	1319 (28.2)	0.011	1922 (28.6)	1910 (28.4)	0.004
Sulfonylureas	449 (7.4)	439 (7.2)	0.006	394 (8.4)	399 (8.5)	0.004	467 (6.9)	440 (6.5)	0.016
Alpha glucosidase inhibitors	10 (0.2)	10 (0.2)	<0.001	10 (0.2)	10 (0.2)	<0.001	10 (0.1)	10 (0.1)	<0.001
Thiazolidinediones	104 (1.7)	104 (1.7)	<0.001	84 (1.8)	81 (1.7)	0.005	109 (1.6)	94 (1.4)	0.018
Insulin	1708 (28.1)	1758 (28.9)	0.018	1481 (31.6)	1561 (33.3)	0.036	1764 (26.2)	1716 (25.5)	0.016
Glucocorticoids	3131 (51.5)	3112 (51.2)	0.021	2323 (49.6)	2245 (47.9)	0.092	3513 (52.2)	3264 (48.5)	0.074
Laboratory									
HbA1c ≥ 9%	610 (10.0)	654 (10.8)	0.024	508 (10.8)	537 (11.5)	0.020	644 (9.6)	579 (8.6)	0.034
BMI ≥25 kg/m^2^	4399 (72.4)	4449 (73.2)	0.018	3175 (67.8)	3235 (69.1)	0.028	4982 (74.1)	4996 (74.3)	0.005

* Values are mean ± SD, or n (%). Any characteristic with a standardized mean difference (SMD) between cohorts <0.10 was considered to be well-matched. GLP-1RA: Glucagon-like peptide-1, receptor agonists; DPP-4i: Dipeptidyl peptidase-4, inhibitors; COPD: chronic obstructive pulmonary disease; HbA1c: Glycated Hemoglobin; BMI: body mass index.

### TZP versus SGLT2i cohort

Compared with the SGLT2i group, TZP use was associated with a significantly lower risk of COPD exacerbation (114.6 vs. 147.0 events per 1000 person-years; HR, 0.73; 95% CI, 0.64–0.82; P < 0.0001; E-value 2.10 [95% LCL, 1.74]; Table 2). Kaplan–Meier curves demonstrated a lower cumulative incidence of COPD exacerbation among TZP users (log-rank P < 0.05; Figure 2A). Subgroup analyses yielded directionally consistent results across all prespecified strata, with HRs <1 in each subgroup (Supplementary Figure S2A). Subgroup HRs for COPD exacerbation ranged from 0.40 (95% CI, 0.26–0.61) in patients with HbA1c ≥ 9% to 0.82 (95% CI, 0.71–0.95) in those with BMI ≥25. An interaction was observed according to baseline HbA1c, with a stronger inverse association among patients with HbA1c ≥ 9% than among those with HbA1c <9% (*P* for interaction = 0.0001; Supplementary Figure S2A).

**TABLE 2:** Hazard ratios for the primary outcome (chronic obstructive pulmonary disease exacerbation) comparing tirzepatide with comparator therapies.

Comparator therapy	Tirzepatide* event no. (IR, 1000PYs)	Comparator event no. (IR, 1000PYs)	HR (95% CI)	*P*-value	E-value (LCL)
SGLT2i (N= 6,080)	422 (114.6)	645 (147.0)	0.73 (0.64, 0.82)	<0.0001	2.10 (1.74)
DPP-4i (N = 4,684)	331 (116.5)	521 (149.0)	0.72 (0.63, 0.82)	<0.0001	2.13 (1.72)
GLP-1RA (N = 6,727)	449 (109.9)	572 (107.6)	0.97 (0.86, 1.10)	0.39	1.21 (1.20)

PYs, person-years; CI, confidence interval; DPP-4i, dipeptidyl peptidase four inhibitor; GLP-1RA, glucagon-like peptide-1, receptor agonist; HR, hazard ratio; LCL, lower confidence limit; SGLT-2i, sodium-glucose cotransporter two inhibitor.

* Propensity score matching (1:1 ratio) was performed separately for each comparison between the tirzepatide group and its respective comparator.

**FIGURE 2:**
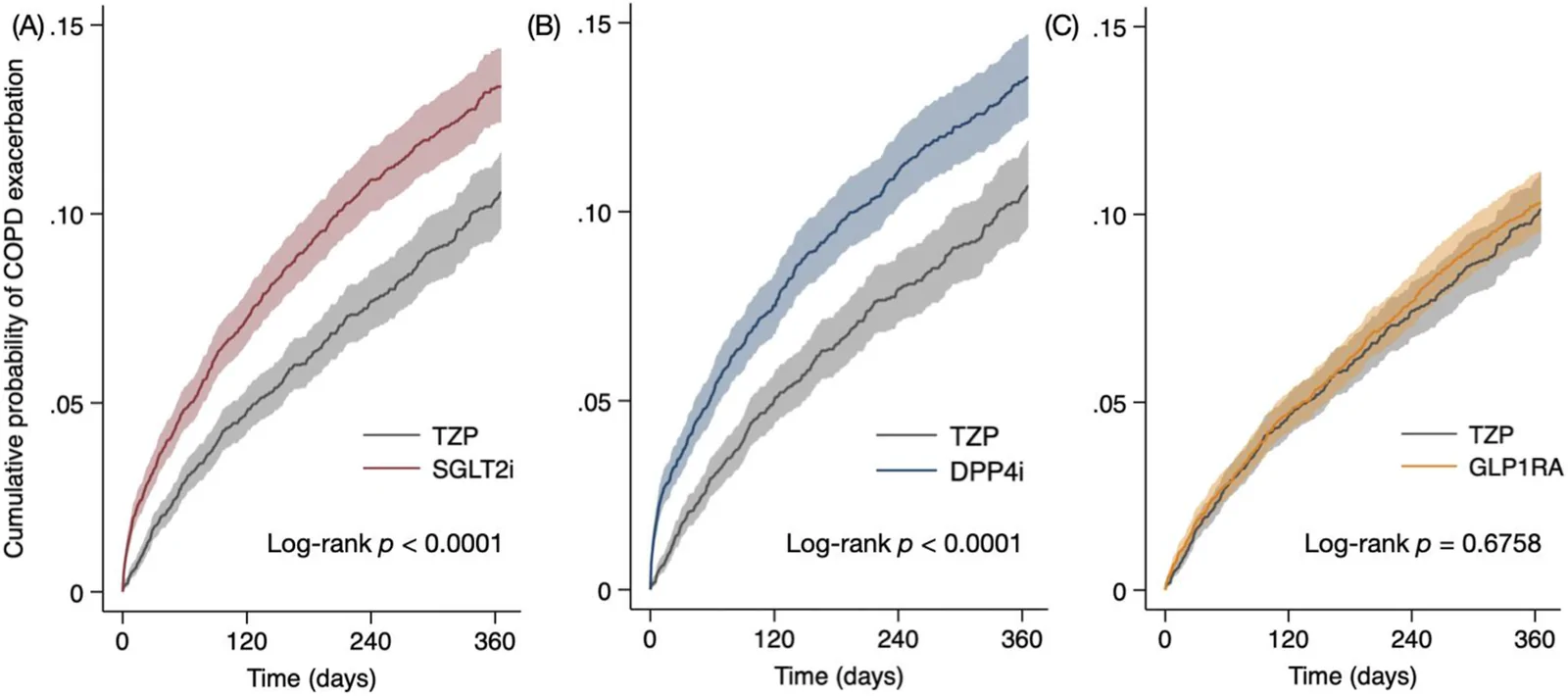
Cumulative incidence of the primary outcome (chronic obstructive pulmonary disease exacerbation) comparing tirzepatide with comparator therapies: **(A)** sodium-glucose cotransporter two inhibitors (SGLT2i); **(B)** dipeptidyl peptidase four inhibitors (DPP-4i); and **(C)** glucagon-like peptide-1 receptor agonists (GLP-1RA).

Regarding secondary outcomes, TZP was also associated with a markedly reduced risk of all-cause mortality (HR, 0.30; 95% CI, 0.23–0.38; P < 0.0001; E-value 6.21 [95% LCL, 4.65]), acute respiratory failure (HR, 0.52; 95% CI, 0.47–0.58; P < 0.0001; E-value 3.24 [95% LCL, 2.85]), and lower respiratory tract infection (HR, 0.72; 95% CI, 0.64–0.80; P < 0.0001; E-value 2.15 [95% LCL, 1.81]; Table 3). Findings for secondary outcomes were consistent across all evaluated subgroups (Supplementary Figures S3A–S5A). Across these strata, HRs ranged from 0.27 to 0.39 for all-cause mortality, 0.50–0.58 for acute respiratory failure, and 0.65–0.80 for lower respiratory tract infection.

**TABLE 3:** Hazard ratios for secondary outcomes comparing tirzepatide with comparator therapies.

Comparator therapy	Tirzepatide* event no. (IR, 1000PYs)	Comparator event no. (IR, 1000PYs)	HR (95% CI)	*P*-value	E-value (LCL)
SGLT2i (N = 6,080)					
All-cause mortality	72 (19.5)	282 (64.3)	0.30 (0.23, 0.38)	<0.0001	6.21 (4.65)
Acute respiratory failure	535 (145.2)	1,047 (238.6)	0.52 (0.47, 0.58)	<0.0001	3.24 (2.85)
Lower respiratory tract infection	512 (139.0)	801 (182.5)	0.72 (0.64, 0.80)	<0.0001	2.15 (1.81)
DPP-4i (N = 4,684)					
All-cause mortality	61 (21.5)	309 (88.4)	0.24 (0.18, 0.31)	<0.0001	7.98 (5.93)
Acute respiratory failure	410 (144.4)	864 (247.2)	0.51 (0.45, 0.57)	<0.0001	3.37 (2.92)
Lower respiratory tract infection	401 (141.2)	765 (218.8)	0.59 (0.52, 0.66)	<0.0001	2.81 (2.40)
GLP-1RA (N = 6,727)					
All-cause mortality	75 (18.4)	177 (33.3)	0.55 (0.42, 0.72)	<0.0001	3.07 (2.15)
Acute respiratory failure	577 (141.3)	729 (137.2)	0.96 (0.86, 1.07)	0.4339	1.26 (1.18)
Lower respiratory tract infection	556 (136.1)	714 (134.4)	0.97 (0.87, 1.09)	0.6114	1.20 (1.19)

IR, incidence rate; PYs, person-years; CI, confidence interval; DPP-4i, dipeptidyl peptidase four inhibitor; GLP-1RA, glucagon-like peptide-1, receptor agonist; HR, hazard ratio; LCL, lower confidence limit; SGLT-2i, sodium-glucose cotransporter two inhibitor.

* Propensity score matching (1:1 ratio) was performed separately for each comparison between the tirzepatide group and its respective comparator.

### TZP versus DPP-4i cohort

Compared with DPP-4i, TZP use was associated with a significantly lower risk of COPD exacerbation (116.5 vs. 149.0 events per 1000 person-years; HR, 0.72; 95% CI, 0.63–0.82; P < 0.0001; E-value 2.13 [95% LCL, 1.72]; Table 2). The Kaplan–Meier curves demonstrated a markedly lower cumulative incidence of COPD exacerbations among TZP users (log-rank P < 0.05; Figure 2B). Subgroup analyses showed directionally consistent results across all prespecified strata, with HRs below one in each category (Supplementary Figure S2B). Subgroup HRs ranged from 0.42 (95% CI, 0.28–0.62) in patients with HbA1c ≥ 9% to 0.75 (95% CI, 0.57–0.99) in those with BMI <25.

For secondary outcomes, TZP treatment was associated with a substantially lower risk of all-cause mortality (HR, 0.24; 95% CI, 0.18–0.31; P < 0.0001; E-value 7.98 [95% LCL, 5.93]), acute respiratory failure (HR, 0.51; 95% CI, 0.45–0.57; P < 0.0001; E-value 3.37 [95% LCL, 2.92]), and lower respiratory tract infection (HR, 0.59; 95% CI, 0.52–0.66; P < 0.0001; E-value 2.81 [95% LCL, 2.40]; Table 3). Associations for secondary outcomes were directionally consistent across all prespecified subgroups (Supplementary Figures S3B–S5B). Across these strata, HRs ranged from 0.17 to 0.30 for all-cause mortality, 0.41–0.54 for acute respiratory failure, and 0.50–0.68 for lower respiratory tract infection.

### TZP versus GLP-1RA cohort

In the comparison with GLP-1RA, the incidence of COPD exacerbation was comparable between groups (109.9 vs. 107.6 events per 1000 person-years; HR, 0.97; 95% CI, 0.86–1.10; P = 0.39; E-value 1.21 [95% LCL, 1.20]; Table 2). Kaplan-Meier analysis showed nearly overlapping cumulative incidence patterns (log-rank P = 0.68; Figure 2C). Subgroup analyses revealed no significant effect modification across most of prespecified strata (Supplementary Figure S2C). A significant difference was observed only among men (HR, 0.80; 95% CI, 0.64–0.99) and those with HbA1c ≥ 9% (HR, 0.52; 95% CI, 0.36–0.77).

For secondary outcomes, TZP use was associated with a significantly lower risk of all-cause mortality (HR, 0.55; 95% CI, 0.42–0.72; P < 0.0001; E-value 3.07 [95% LCL, 2.15]). In contrast, no significant differences were observed between TZP and GLP-1RA in the risks of acute respiratory failure (HR, 0.96; 95% CI, 0.86–1.07; P = 0.43; E-value 1.26 [95% LCL, 1.18]) or lower respiratory tract infection (HR, 0.97; 95% CI, 0.87–1.09; P = 0.61; E-value 1.20 [95% LCL, 1.19]; Table 3).

Subgroup analysis of all-cause mortality demonstrated a consistent association across all prespecified subgroups (Supplementary Figure S3C). For acute respiratory failure, risk estimates were also consistent with statistically significant associations observed in patients aged ≥65 years, men, those without recent COPD exacerbations, and individuals with BMI <25 (Supplementary Figure S4C). Similarly, analyses of lower respiratory tract infection demonstrated broadly consistent trends across subgroups, except in patients with BMI ≥25, in whom the association showed an opposite direction compared with the overall estimate (Supplementary Figure S5C).

### Absolute effect estimates

Absolute differences in Kaplan–Meier estimated cumulative incidence at day 365 generally supported the primary analyses (Supplementary Table S7). For COPD exacerbation, the absolute differences were 2.80 and 2.88 percentage points for TZP versus SGLT2i and DPP-4i, corresponding to descriptive NNT-equivalent estimates of 36 and 35, respectively. The absolute difference between TZP and other GLP-1RAs was minimal (0.18 percentage points), and no NNT-equivalent estimate was reported because the association was not statistically significant. Absolute estimates for secondary outcomes are also provided in Supplementary Table S7. For all-cause mortality, the NNT-equivalent estimates were 25 for TZP vs. SGLT2i, 17 for TZP vs. DPP-4i, and 72 for TZP vs. other GLP-1RA. For acute respiratory failure, the NNT-equivalent estimates were 12 for both TZP vs. SGLT2i and TZP vs. DPP-4i. For lower respiratory tract infection, the NNT-equivalent estimates were 29 for TZP vs. SGLT2i and 16 for TZP vs. DPP-4i. No NNT-equivalent estimate was reported for acute respiratory failure or lower respiratory tract infection because the associations were not statistically significant for TZP vs. other GLP-1RA.

### Negative outcome analyses

No statistically significant associations were observed between TZP use and the risks of skin cancer or cataract across all comparator cohorts (skin cancer: HR 0.88 [95% CI 0.62–1.24] for TZP vs. SGLT2i, HR 0.70 [95% CI 0.48–1.02] for TZP vs. DPP-4i, and HR 1.00 [95% CI 0.71–1.40] for TZP vs. GLP-1RA; cataract: HR 1.03 [95% CI 0.78–1.37] for TZP vs. SGLT2i, HR 1.15 [95% CI 0.85–1.59] for TZP vs. DPP-4i, and HR 0.97 [95% CI 0.75–1.25] for TZP vs. GLP-1RA) (Supplementary Table S8).

## Discussion

In this large, real-world cohort of patients with coexisting COPD and T2D, we evaluate the comparative effectiveness of TZP versus other commonly prescribed glucose-lowering agents in reducing the risk of COPD exacerbations and all-cause mortality. We found that TZP was associated with a 27% lower risk compared with SGLT2i and a 28% lower risk of COPD exacerbations compared with DPP-4i. TZP also conferred substantial reductions in all-cause mortality - 70% lower than SGLT2i and 76% lower than DPP-4i. Additionally, TZP was associated with a 48% lower risk of acute respiratory failure and a 18% lower risk of lower respiratory tract infection compared with SGLT2i, and with 49% and 41% reductions in those outcomes, respectively, compared with DPP-4i. These findings suggest that, compared with SGLT2i or DPP-4i, the dual GIP and GLP-1 receptor activity of TZP may be associated with clinically meaningful respiratory and survival benefits in patients with COPD and T2D. TZP may therefore help address multiple therapeutic objectives simultaneously, potentially reducing disease burden and simplifying treatment regimens. However, randomized clinical trials are warranted to confirm these observations and elucidate underlying mechanisms.

Our findings extend a growing body of observational evidence on incretin-based therapies and COPD outcomes (Pirera et al., 2025; Yen et al., 2024; Wu et al., 2026). A cohort study of GLP-1RA reported a 46% lower risk of all-cause mortality and a lower risk of adverse cardiopulmonary outcomes compared with non-GLP-1RA therapy in patients with T2D and COPD (Yen et al., 2024). Similarly, a systematic review and network meta-analysis comparing SGLT2i, GLP-1RA, DPP-4i, and sulfonylureas found that GLP-1RA and SGLT2i, but not DPP-4i, were associated with a significantly lower risk of moderate-to-severe COPD exacerbations among patients with T2D (Pirera et al., 2025). Our findings are directionally concordant with this literature, although the magnitude of the mortality reduction associated with TZP is considerably larger than previously reported for GLP-1RA as a class, which we address further below.

The magnitude of the mortality reduction associated with TZP in this study is larger than what has typically been reported for glucose-lowering or incretin-based therapies in cardiovascular and metabolic outcome trials. This may partly reflect TZP’s dual GIP/GLP-1 receptor agonism, which is associated with greater weight loss and glycemic improvement than SGLT2i, DPP-4i, or single-agonist GLP-1RAs, and this may be relevant to the larger reductions in cardiometabolic and respiratory mortality observed compared with older agents. Healthy user bias and confounding by indication likely also played a role, since patients selected for TZP, a newer and more costly self-injectable agent, may differ systematically from those prescribed established generic therapies in ways our matching variables did not fully capture. Given this, an effect of this size should be viewed as hypothesis-generating pending confirmation in prospective or randomized studies.

The observed reductions in COPD exacerbations and mortality with TZP, compared with SGLT2i and DPP-4i, may reflect the complex effects of these therapies, although the underlying mechanisms cannot be determined from our observational data. Both GIP and GLP-1 receptor signaling have been reported to modulate systemic and pulmonary inflammation through distinct pathways (Lee and June 2016; Drucker, 2016; Toki et al., 2024; Wang et al., 2024; Cai et al., 2018), which could potentially contribute to the observed associations. However, this interpretation remains speculative and requires further investigation. In addition, the effects of TZP on weight reduction and glycemic control may confer cardiometabolic benefits that could be relevant to the systemic inflammatory burden associated with COPD progression (Borlaug et al., 2025). Together, these potential mechanisms provide possible explanations for the observed associations and warrant further investigation in prospective studies.

Compared with GLP-1RA, TZP was associated with similar risks of COPD exacerbation and other respiratory outcomes, including acute respiratory failure and lower respiratory tract infection. These findings suggest that the respiratory outcomes associated with TZP may be broadly comparable to those of single-agonist GLP-1RAs. The similarity may be consistent with a potential contribution of shared GLP-1 receptor activity, although this hypothesis cannot be established from our observational data. TZP was associated with a lower risk of all-cause mortality than GLP-1RA; however, the reasons for this difference remain uncertain. Differences in glycemic control, weight reduction, or other metabolic effects may potentially contribute and warrant further investigation (Aronne et al., 2025; Frías et al., 2021; Jastreboff et al., 2025; Finan et al., 2013). Given the observational design, these findings should be interpreted as associations rather than evidence of causal or mechanistic effects. Prospective studies are needed to further clarify the comparative cardiometabolic and respiratory effects of TZP and GLP-1RA in patients with COPD and T2D.

This study has several notable strengths. First, it utilizes a large, geographically diverse, real-world population from the TriNetX global network, encompassing multiple healthcare systems across different countries, thereby enhancing external validity and generalizability to diverse clinical settings. The substantial sample size provided robust statistical power to detect clinically meaningful differences in both primary and secondary outcomes. Second, rigorous 1:1 propensity score matching was employed to minimize confounding by indication and selection bias, achieving excellent covariate balance (all standardized mean differences <0.1) across numerous baseline characteristics including demographics, comorbidities, and concomitant medications. Third, The use of an active-comparator, new-user design, comparing TZP with other contemporary glucose-lowering agents rather than placebo or no treatment, improved comparability between treatment groups, and reduced the potential for immortal time bias. Fourth, the inclusion of multiple active comparators (SGLT2i, DPP-4i, and GLP-1RA) with distinct mechanisms of action enabled comprehensive assessment of TZP’s comparative effectiveness across the current therapeutic landscape. Fifth, the consistency of treatment effects across primary and secondary outcomes, combined with high E-values (ranging from 2.10 to 7.98), strengthens the robustness of findings against potential unmeasured confounding. Sixth, the inclusion of prespecified negative outcome controls (skin cancer and cataract) provides additional evidence against residual confounding; the absence of associations between TZP and these outcomes, which are not biologically plausible to be affected by incretin-based therapy, supports the specificity of observed effects on respiratory and mortality outcomes and suggests that baseline differences between treatment groups were adequately controlled through propensity score matching. Finally, prespecified stratified analyses across clinically relevant subgroups demonstrated consistent associations, supporting the generalizability of findings across different patient populations and identifying potential effect modifiers.

This study has several limitations that warrant consideration. First, as a retrospective observational analysis using real-world data, the potential for residual confounding cannot be fully excluded despite rigorous propensity score matching. Unmeasured or inadequately measured confounders may have influenced treatment assignment and outcomes. In particular, granular measures of COPD severity, including GOLD stage, spirometric indices (FEV1, FEV1/FVC ratio), CAT score, and long-term oxygen therapy status—were not available in the TriNetX network, and residual confounding by disease severity is likely despite adjustment for baseline exacerbation frequency. Smoking intensity (pack-years) and current smoking status were similarly unavailable, and smoking-related confounding cannot be excluded. Socioeconomic factors were captured only through ICD-based proxies, which may inadequately reflect true socioeconomic status. Second, medication exposure was ascertained from electronic prescription records rather than pharmacy dispensing data or direct patient reports, and information on medication dose was not available. This may not accurately reflect actual medication use, adherence, dosing intensity, or treatment discontinuation, and could bias effect estimates toward the null if non-differential, or in either direction if it varied systematically between groups. Third, outcomes were identified using ICD-10-CM diagnostic codes, which are subject to coding inaccuracies, underreporting, and delayed data entry. Exacerbation severity (e.g., mild versus requiring hospitalization) could not be reliably distinguished, and the absence of standardized clinical adjudication may have resulted in outcome misclassification. Fourth, the relatively short follow-up duration limited assessment of long-term respiratory outcomes, late cardiovascular events, and durability of treatment effect. Fifth, the study population was drawn from healthcare organizations contributing to TriNetX and may not be fully representative of all patients with COPD and T2D, particularly in underserved settings or countries with different healthcare systems. Sixth, changes in concomitant therapy during follow-up, including adjustments to inhaled medications, corticosteroid use, or other glucose-lowering agents, could not be accounted for and may have modified treatment effects. Seventh, indication bias may persist despite matching, as clinicians may preferentially prescribe newer agents such as TZP to patients perceived as healthier or more treatment-adherent. Finally, the TriNetX platform provides aggregated, cohort-level outputs rather than exportable patient-level data. Matched-pair identifiers were also unavailable; therefore, the standard errors from the Cox models could not account for within-pair correlation, and the statistical analysis did not fully account for the matched design. This limitation may have affected the precision of the estimated hazard ratios and their confidence intervals and should be considered when interpreting the results. The lack of patient-level data also precluded inverse probability weighting, overlap weighting, evaluation of alternative caliper specifications, formal multiplicity correction across subgroup and secondary analyses, and presentation of numbers at risk beneath the Kaplan–Meier curves. To partially address these constraints, we performed a negative control outcome analysis and interpreted subgroup findings as hypothesis-generating rather than confirmatory.

## Conclusion

Among patients with coexisting COPD and T2D, TZP was associated with a lower risk of COPD exacerbations and respiratory complications compared with SGLT2i and DPP-4i, and a lower risk of all-cause mortality across all comparator groups, including GLP-1RA. Prospective studies and randomized clinical trials are warranted to confirm these associations and to further elucidate the mechanisms linking incretin-based therapies to respiratory protection.

## Data Availability

The original contributions presented in the study are included in the article/Supplementary Material, further inquiries can be directed to the corresponding author.
