# Supplementary material for: Tirzepatide versus other glucose-lowering therapies and risk of COPD exacerbations in patients with comorbid type 2 diabetes: a retrospective cohort study
Source: Front Pharmacol. 2026 Oct 9;17:1917340. doi: 10.3389/fphar.2026.1917340 (PMC13690786; doi:10.3389/fphar.2026.1917340)
Supplement: Supplementary file 1 [file DataSheet1.docx]

**Supplementary Material**

**Tirzepatide versus Other Glucose-Lowering Therapies and Risk of COPD Exacerbations in Patients with Comorbid Type 2 Diabetes: A Retrospective Cohort Study**

Wan-Hsuan Hsu^1^, Yang-En Chan^2^, Jheng-Yan Wu^3,4^, Yao-Ming Chuang^2^, Chia-Yu Kuo^2^, Bo-Wen Shiau^1^, Po-Yu Huang^1^, Chih-Cheng Lai^5,6*^

^1^Department of Internal Medicine, Chi Mei Medical Center, Taiwan

^2^Department of General Medicine, Chi Mei Medical Center, Tainan, Taiwan

^3^Department of Nutrition, Chi Mei Medical Center, Tainan, Taiwan

^4^Department of Public Health, College of Medicine, National Cheng Kung University, Tainan, Taiwan

^5^Department of Intensive Care Medicine, Chi Mei Medical Center, Tainan, Taiwan.

^6^School of Medicine, College of Medicine, National Sun Yat-sen University, Kaohsiung, Taiwan

***Corresponding author:**

Chih-Cheng Lai, Department of Intensive Care Medicine, Chi Mei Medical Center, Tainan, Taiwan and School of Medicine, College of Medicine, National Sun Yat-sen University, Kaohsiung, Taiwan. Email: [dtmed141@gmail.com](mailto:dtmed141@gmail.com)

Word count

Abstract: 234

Text: 3299

**Supplementary Figure 1. Study timeline for each pairwise active-comparator cohort.**

Eligible index dates occurred between June 1, 2022, and May 30, 2025. For each pairwise comparison, the pre-index washout period extended from day −365 through day −1. The index date was defined as the date of the first qualifying prescription of tirzepatide or the respective comparator. Outcomes were assessed from day 1 through day 365 after the index date. Post-index medication use was not used to determine cohort eligibility.

**Supplementary Figure 2. Subgroup analyses of the primary outcome (chronic obstructive pulmonary disease exacerbation) comparing tirzepatide with (A) sodium-glucose cotransporter 2 inhibitors (SGLT2i); (B) dipeptidyl peptidase 4 inhibitors (DPP-4i); and (C) glucagon-like peptide-1 receptor agonists (GLP-1RA).**

**Supplementary Figure 3. Subgroup analyses of secondary outcome (all-cause mortality) comparing tirzepatide with (A) sodium-glucose cotransporter 2 inhibitors (SGLT2i); (B) dipeptidyl peptidase 4 inhibitors (DPP-4i); and (C) glucagon-like peptide-1 receptor agonists (GLP-1RA).**

**Supplementary Figure 4. Subgroup analyses of secondary outcome (acute respiratory failure) comparing tirzepatide with (A) sodium-glucose cotransporter 2 inhibitors (SGLT2i); B) dipeptidyl peptidase 4 inhibitors (DPP-4i); and (C) glucagon-like peptide-1 receptor agonists (GLP-1RA).**

**Supplementary Figure 5. Subgroup analyses of secondary outcome (lower respiratory tract infection) comparing tirzepatide with (A) sodium-glucose cotransporter 2 inhibitors (SGLT2i); B) dipeptidyl peptidase 4 inhibitors (DPP-4i); and (C) glucagon-like peptide-1 receptor agonists (GLP-1RA).**

**Supplementary Table 1. Demographic, diagnostic, procedural, medication, visit, and laboratory codes used to define the study cohorts.**

| **Category** | **Code** | | **Description** | |
| --- | --- | --- | --- | --- |
| **#1 Age at index and HCO compliance** | |  | |  |
| demographics | Age | | Age (at least 40 years) | |
| visit | TNX:Visit | | Visit (greater than or equal to 3 instances) | |
| **#2 Patients with COPD and type 2 diabetes mellitus** | | | |  |
| (must have #2.1 and any of #2.2) | |  | |  |
| #2.1 Chronic obstructive pulmonary disease (COPD) | | | |  |
| diagnosis | UMLS:ICD10CM:J41 | | Simple and mucopurulent chronic bronchitis | |
| diagnosis | UMLS:ICD10CM:J42 | | Unspecified chronic bronchitis | |
| diagnosis | UMLS:ICD10CM:J43 | | Emphysema | |
| diagnosis | UMLS:ICD10CM:J44 | | Other chronic obstructive pulmonary disease | |
| #2.2 Type 2 diabetes mellitus | |  | |  |
| diagnosis | UMLS:ICD10CM:E11 | | Type 2 diabetes mellitus | |
| laboratory | TNX:9037 | | Hemoglobin A1c/Hemoglobin.total in Blood (at least 6.50 %) | |
| **#3 Exclusion criteria** | |  | |  |
| Definition of index date: date received study medications | | | |  |
| (must NOT have any of the following prior to the index date) | | | |  |
| diagnosis | UMLS:ICD10CM:E10 | | Type 1 diabetes mellitus | |
| diagnosis | UMLS:ICD10CM:E13 | | Other specified diabetes mellitus | |
| diagnosis | UMLS:ICD10CM:O24.4 | | Gestational diabetes mellitus | |
| **#4 Cohort definition** | |  | |  |
| **#4.1 TZP group** | |  | |  |
| (must have the following and must NOT have #4.2, #4.3 and #4.4 during the 365 days before the index date) | | | |  |
| medication | NLM:RXNORM:2601723 | | tirzepatide | |
| **#4.2GLP-1RA group** | |  | |  |
| (must have any of the following and must NOT have #4.1, #4.3 and #4.4 during the 365 days before the index date) | | | |  |
| medication | NLM:RXNORM:1534763 | | albiglutide | |
| medication | NLM:RXNORM:1551291 | | dulaglutide | |
| medication | NLM:RXNORM:1991302 | | semaglutide | |
| medication | NLM:RXNORM:475968 | | liraglutide | |
| medication | NLM:RXNORM:60548 | | exenatide | |
| **#4.3 DPP-4i group** | |  | |  |
| (must have any of the following and must NOT have #4.1, #4.2 and #4.4 during the 365 days before the index date) | | | |  |
| medication | NLM:ATC:A10BH | | Dipeptidyl peptidase 4 (DPP-4) inhibitors | |
| medication | NLM:RXNORM:1100699 | | linagliptin | |
| medication | NLM:RXNORM:1368001 | | alogliptin | |
| medication | NLM:RXNORM:593411 | | sitagliptin | |
| medication | NLM:RXNORM:596554 | | vildagliptin | |
| medication | NLM:RXNORM:857974 | | saxagliptin | |
| **#4.4 SGLT2i group** | |  | |  |
| (must have any of the following and must NOT have #4.1, #4.2 and #4.3 during the 365 days before the index date) | | | |  |
| medication | NLM:ATC:A10BK | | Sodium-glucose co-transporter 2 (SGLT2) inhibitors | |
| medication | NLM:RXNORM:1373458 | | canagliflozin | |
| medication | NLM:RXNORM:1488564 | | dapagliflozin | |
| medication | NLM:RXNORM:1545653 | | empagliflozin | |
| medication | NLM:RXNORM:1992672 | | ertugliflozin | |
| medication | NLM:RXNORM:2627044 | | bexagliflozin | |
| medication | NLM:RXNORM:2638675 | | sotagliflozin | |

**Supplementary Table 2. Demographic, diagnostic, and laboratory codes used to define covariates.**

| **Category** | **Code** | **Description** |
| --- | --- | --- |
| Demographics | AI | Age at Index |
| Demographics | F | Female |
| Demographics | M | Male |
| Demographics | 56370 | Black or African American |
| Demographics | 75301 | White |
| Demographics | 1002-5 | American Indian or Alaska Native |
| Demographics | UNK | Unknown Race |
| Demographics | 64498 | Native Hawaiian or Other Pacific Islander |
| Demographics | UN | Unknown Ethnicity |
| Demographics | 104582 | Not Hispanic or Latino |
| Demographics | 85865 | Hispanic or Latino |
| Demographics | 84373 | Other Race |
| Demographics | 46997 | Asian |
| Diagnosis | I10-I1A | Hypertensive diseases |
| Diagnosis | I10-I15 | Hypertensive diseases (deprecated 2018) |
| Diagnosis | I20-I25 | Ischemic heart diseases |
| Diagnosis | I60-I69 | Cerebrovascular diseases |
| Diagnosis | I26-I28 | Pulmonary heart disease and diseases of pulmonary circulation |
| Diagnosis | J44.1 | Chronic obstructive pulmonary disease with (acute) exacerbation |
| Diagnosis | K74 | Fibrosis and cirrhosis of liver |
| Diagnosis | E08-E13 | Diabetes mellitus |
| Diagnosis | C34 | Malignant neoplasm of bronchus and lung |
| Diagnosis | N18.3 | Chronic kidney disease, stage 3 (moderate) |
| Diagnosis | N18.4 | Chronic kidney disease, stage 4 (severe) |
| Diagnosis | N18.6 | End stage renal disease |
| Diagnosis | F20-F29 | Schizophrenia, schizotypal, delusional, and other non-mood psychotic disorders |
| Diagnosis | F30-F39 | Mood [affective] disorders |
| Diagnosis | Z55-Z65 | Persons with potential health hazards related to socioeconomic and psychosocial circumstances |
| Diagnosis | F17 | Nicotine dependence |
| Diagnosis | Z87.891 | Personal history of nicotine dependence |
| Diagnosis | G47.33 | Obstructive sleep apnea (adult) (pediatric) |
| Diagnosis | Z95.0 | Presence of cardiac pacemaker |
| Diagnosis | E66 | Overweight and obesity |
| Medication | A10A | INSULINS AND ANALOGUES |
| Medication | A10BA | Biguanides |
| Medication | A10BB | Sulfonylureas |
| Medication | A10BF | Alpha glucosidase inhibitors |
| Medication | A10BG | Thiazolidinediones |
| Laboratory | 9083 | BMI ≥ 30kg/m2 |
| Laboratory | 9037 | Hemoglobin A1c/Hemoglobin.total in Blood ≥ 9% |

**Supplementary Table 3. Demographic, diagnostic, and procedural codes used to define study outcomes.**

| Index date: date received the study medications | | |  | | |
| --- | --- | --- | --- | --- | --- |
| Outcome time window: 1 day to 1 year after the index date | | | | |  |
| **Category** | **Code** | **Description** | |  |  |
| **#1 Primary outcome: COPD exacerbation** | | | |  |  |
| Diagnosis | UMLS:ICD10CM:J44.1 | Chronic obstructive pulmonary disease with (acute) exacerbation | |  |  |
| **#2 Secondary outcomes** | |  | |  |  |
| **#2.1 All-cause mortality** | |  | |  |  |
| Demographics | Deceased | Deceased | |  |  |
| **#2.2 Acute respiratory failure** | |  | |  |  |
| Diagnosis | UMLS:ICD10CM:J96.0 | Acute respiratory failure | |  |  |
| Procedure | UMLS:SNOMED:112798008 | Insertion of endotracheal tube | |  |  |
| Procedure | UMLS:SNOMED:1149092001 | Positive pressure ventilation | |  |  |
| Procedure | UMLS:CPT:31500 | Intubation, endotracheal, emergency procedure | |  |  |
| Procedure | UMLS:ICD10PCS:5A1935Z | Respiratory Ventilation, Less than 24 Consecutive Hours | |  |  |
| Procedure | UMLS:ICD10PCS:5A1945Z | Respiratory Ventilation, 24-96 Consecutive Hours | |  |  |
| Procedure | UMLS:ICD10PCS:5A1955Z | Respiratory Ventilation, Greater than 96 Consecutive Hours | |  |  |
| Procedure | UMLS:ICD10PCS:5A19 | Physiological Systems / Performance / Respiratory | |  |  |
| Procedure | UMLS:ICD10PCS:5A09357 | Assistance with Respiratory Ventilation, Less than 24 Consecutive Hours, Continuous Positive Airway Pressure | |  |  |
| Procedure | UMLS:ICD10PCS:5A09358 | Assistance with Respiratory Ventilation, Less than 24 Consecutive Hours, Intermittent Positive Airway Pressure | |  |  |
| Procedure | UMLS:ICD10PCS:5A09359 | Assistance with Respiratory Ventilation, Less than 24 Consecutive Hours, Continuous Negative Airway Pressure | |  |  |
| Procedure | UMLS:ICD10PCS:5A0935A | Assistance with Respiratory Ventilation, Less than 24 Consecutive Hours, High Flow/Velocity Cannula | |  |  |
| Procedure | UMLS:ICD10PCS:5A0935A | Assistance with Respiratory Ventilation, Less than 24 Consecutive Hours, High Flow/Velocity Cannula | |  |  |
| Procedure | UMLS:ICD10PCS:5A0935B | Assistance with Respiratory Ventilation, Less than 24 Consecutive Hours, Intermittent Negative Airway Pressure | |  |  |
| Procedure | UMLS:ICD10PCS:5A09457 | Assistance with Respiratory Ventilation, 24-96 Consecutive Hours, Continuous Positive Airway Pressure | |  |  |
| Procedure | UMLS:ICD10PCS:5A09458 | Assistance with Respiratory Ventilation, 24-96 Consecutive Hours, Intermittent Positive Airway Pressure | |  |  |
| Procedure | UMLS:ICD10PCS:5A09459 | Assistance with Respiratory Ventilation, 24-96 Consecutive Hours, Continuous Negative Airway Pressure | |  |  |
| Procedure | UMLS:ICD10PCS:5A0945A | Assistance with Respiratory Ventilation, 24-96 Consecutive Hours, High Flow/Velocity Cannula | |  |  |
| Procedure | UMLS:ICD10PCS:5A0945B | Assistance with Respiratory Ventilation, 24-96 Consecutive Hours, Intermittent Negative Airway Pressure | |  |  |
| Procedure | UMLS:ICD10PCS:5A0945Z | Assistance with Respiratory Ventilation, 24-96 Consecutive Hours | |  |  |
| Procedure | UMLS:ICD10PCS:5A09557 | Assistance with Respiratory Ventilation, Greater than 96 Consecutive Hours, Continuous Positive Airway Pressure | |  |  |
| Procedure | UMLS:ICD10PCS:5A09558 | Assistance with Respiratory Ventilation, Greater than 96 Consecutive Hours, Intermittent Positive Airway Pressure | |  |  |
| Procedure | UMLS:ICD10PCS:5A09559 | Assistance with Respiratory Ventilation, Greater than 96 Consecutive Hours, Continuous Negative Airway Pressure | |  |  |
| Procedure | UMLS:ICD10PCS:5A0955A | Assistance with Respiratory Ventilation, Greater than 96 Consecutive Hours, High Flow/Velocity Cannula | |  |  |
| Procedure | UMLS:ICD10PCS:5A0955B | Assistance with Respiratory Ventilation, Greater than 96 Consecutive Hours, Intermittent Negative Airway Pressure | |  |  |
| Procedure | UMLS:ICD10PCS:5A0955Z | Assistance with Respiratory Ventilation, Greater than 96 Consecutive Hours | |  |  |
| Procedure | UMLS:ICD10PCS:0BH1 | Respiratory System / Insertion / Trachea | |  |  |
| **#2.3 Lower respiratory tract infection** | |  | |  |  |
| Diagnosis | UMLS:ICD10CM:J09-J18 | Influenza and pneumonia | |  |  |
| Diagnosis | UMLS:ICD10CM:J20-J22 | Other acute lower respiratory infections | |  |  |
| Diagnosis | UMLS:ICD10CM:J44.0 | Chronic obstructive pulmonary disease with (acute) lower respiratory infection | |  |  |
| **#3 Negative outcomes** | |  | |  |  |
| #3.1 Skin cancer | |  | |  |  |
| Diagnosis | UMLS:ICD10CM:C44 | Other and unspecified malignant neoplasm of skin | |  |  |
| #3.2 Cataract |  |  | |  |  |
| Diagnosis | UMLS:ICD10CM:H26 | Other cataract | |  |  |

**Supplementary Table 4. Codes used for subgroup definitions.**

| Index date: date received the study medications | | | | | |
| --- | --- | --- | --- | --- | --- |
| **Category** | **Code** | | **Description** | | |
| **#1 Age at index** | | |  | | |
| **#1.1 Age 40-64** | | |  | | |
| demographics | Age | | between 40 and 64 years | | |
| **#1.2 Age ≥ 65** | | |  | | |
| demographics | Age | | at least ≥ 65 years | | |
| **#2 Sex** |  | |  | | |
| **#2.1 Female** |  | |  | | |
| demographics | UMLS:HL7V3.0:Gender:F | | Female | | |
| **#2.2 Male** |  | |  | | |
| demographics | UMLS:HL7V3.0:Gender:M | | Male | | |
| **#3 Recent acute exacerbation of COPD** | | | | | |
| **#3.1 Absence** | | | | |  |
| (must NOT have the following within one year prior to the index date) | | | | | |
| Diagnosis | | UMLS:ICD10CM:J44.1 | | Chronic obstructive pulmonary disease with (acute) exacerbation | |
| **#3.2 Presence** | | | | |  |
| (must have #3.1 within one year prior to the index date) | | | | | |
| **#4 DM control** |  | | | |  |
| **#4.1 HbA1c < 9%** | | | | |  |
| (must have the following on or before index date) | | | | | |
| Laboratory | 9037 | | | | Hemoglobin A1c/Hemoglobin.total in Blood (less than 9%) |
| (must *not* have the following on or before index date) | | | | | |
| Laboratory | 9037 | | | | Hemoglobin A1c/Hemoglobin.total in Blood (at least 9%) |
| **#4.2 HbA1c ≥ 9%** | | | | |  |
| (must have the following on or before index date) | | | | | |
| Laboratory | 9037 | | | | Hemoglobin A1c/Hemoglobin.total in Blood (at least 9%) |
| **#5 Body mass index (BMI)** | | | | |  |
| **#5.1 BMI <25** | | | | |  |
| (must have the following on or before index date) | | | | | |
| laboratory | TNX:9083 | | | | BMI (less than 25 kg/m2) |
| (must *not* have the following on or before index date) | | | | | |
| laboratory | TNX:9083 | | | | BMI (at least 25.00 kg/m2) |
| **#5.2 BMI ≥25** | | | | |  |
| (must have the following on or before index date) | | | | | |
| laboratory | TNX:9083 | | | | BMI (at least 25.00 kg/m2) |

**Supplementary Table 5. Follow-up characteristics of the propensity score–matched cohorts.**

| **Comparison** | **Treatment cohort** | **n** | **Mean follow-up, days** | **Median follow-up, days** | **IQR, days** | **Total follow-up, person-years** |
| --- | --- | --- | --- | --- | --- | --- |
| TZP vs SGLT2i | TZP | 6,080 | 221.13 | 232 | 273 | 3,683 |
|  | SGLT2i | 6,080 | 263.43 | 365 | 213.5 | 4,388 |
| TZP vs DPP-4i | TZP | 4,684 | 221.32 | 235 | 275 | 2,840 |
|  | DPP-4i | 4,684 | 272.40 | 365 | 190.5 | 3,496 |
| TZP vs other GLP-1RAs | TZP | 6,727 | 221.64 | 233 | 273 | 4,085 |
|  | Other GLP-1RAs | 6,727 | 288.34 | 365 | 147 | 5,314 |

DPP-4i, dipeptidyl peptidase-4 inhibitor; GLP-1RA, glucagon-like peptide-1 receptor agonist; IQR, interquartile range; SGLT2i, sodium–glucose cotransporter-2 inhibitor; TZP, tirzepatide.

Follow-up was assessed during the prespecified observation period from day 1 through day 365 after the index date. Total person-years were calculated by multiplying the number of patients in each cohort by the corresponding unrounded mean follow-up duration and dividing by 365. The interquartile range represents the difference between the 75th and 25th percentiles, as provided by the TriNetX Analytics platform. Propensity score matching at a 1:1 ratio was performed separately for each comparison.

**Supplementary Table 6. Pre-matching baseline characteristics of the tirzepatide (TZP) cohort and comparator cohorts.**

|  | **TZP group (n=6,728)** | **SGLT2i group (n=59,850)** | **SMD** | **DPP-4i group (n=22,922)** | **SMD** | **GLP-1RA group (n=39,623)** | **SMD** |
| --- | --- | --- | --- | --- | --- | --- | --- |
| **Demographics (%)** |  |  |  |  |  |  |  |
| Age at Index (mean±SD) | 64.3 ± 9.6 | 70.2 ± 10.1 | 0.602 | 73.0 ± 10.5 | 0.868 | 65.0 ± 10.0 | 0.069 |
| *Sex* |  |  |  |  |  |  |  |
| Female | 4131 (61.4) | 24105 (40.3) | 0.432 | 11007 (48.0) | 0.271 | 23775(60) | 0.029 |
| Male | 2593 (38.5) | 35707 (59.7) | 0.432 | 11909 (52.0) | 0.272 | 15835(40) | 0.029 |
| *Race* |  |  |  |  |  |  |  |
| White | 5263 (78.2) | 40518 (67.7) | 0.239 | 11274 (49.2) | 0.634 | 28454(71.8) | 0.149 |
| Black or African American | 957 (14.2) | 11477 (19.2) | 0.133 | 3234 (14.1) | 0.003 | 7173(18.1) | 0.106 |
| Other Race | 185 (2.7) | 1481 (2.5) | 0.017 | 613 (2.7) | 0.004 | 1230(3.1) | 0.021 |
| Unknown Race | 167 (2.5) | 3776 (6.3) | 0.188 | 5260 (22.9) | 0.646 | 1536(3.9) | 0.080 |
| Asian | 70 (1.0) | 1796 (3.0) | 0.140 | 2301 (10.0) | 0.401 | 660(1.7) | 0.054 |
| American Indian or Alaska Native | 44 (0.7) | 434 (0.7) | 0.009 | 108 (0.5) | 0.024 | 310(0.8) | 0.015 |
| Native Hawaiian or Other Pacific. Islander | 42 (0.6) | 368 (0.6) | 0.001 | 132 (0.6) | 0.006 | 260(0.7) | 0.004 |
| *Ethnicity* |  |  |  |  |  |  |  |
| Hispanic or Latino | 313 (4.7) | 2592 (4.3) | 0.016 | 1005 (4.4) | 0.013 | 2259(5.7) | 0.047 |
| Not Hispanic or Latino | 5055 (75.1) | 44286 (74.0) | 0.026 | 13669 (59.6) | 0.336 | 29292(73.9) | 0.028 |
| Unknown Ethnicity | 1360 (20.2) | 12972 (21.7) | 0.036 | 8248 (36) | 0.357 | 8072(20.4) | 0.004 |
| *Lifestyle* |  |  |  |  |  |  |  |
| Nicotine dependence | 1366 (20.3) | 17165 (28.7) | 0.196 | 3815 (16.6) | 0.094 | 9150(23.1) | 0.068 |
| Personal history of nicotine dependence | 1913 (28.4) | 22277 (37.2) | 0.188 | 4773 (20.8) | 0.177 | 10872(27.4) | 0.022 |
| Problem with socioeconomic circumstances | 317 (4.7) | 4082 (6.8) | 0.091 | 921 (4.0) | 0.034 | 1787(4.5) | 0.010 |
| **Comorbid conditions (%)** |  |  |  |  |  |  |  |
| COPD exacerbation | 744 (11.1) | 13276 (22.2) | 0.302 | 2951 (12.9) | 0.056 | 4514(11.4) | 0.011 |
| *Metabolic disorders* |  |  |  |  |  |  |  |
| Diabetes mellitus | 4813 (71.5) | 51182 (85.5) | 0.345 | 19012 (82.9) | 0.274 | 32000(80.8) | 0.218 |
| Overweight and obesity | 3481 (51.7) | 21352 (35.7) | 0.328 | 4488 (19.6) | 0.712 | 18035(45.5) | 0.125 |
| *Cardiovascular diseases* |  |  |  |  |  |  |  |
| Hypertensive diseases | 4767 (70.9) | 50357 (84.1) | 0.322 | 16284 (71.0) | 0.004 | 29259(73.8) | 0.067 |
| Ischemic heart diseases | 1820 (27.1) | 34482 (57.6) | 0.650 | 7864 (34.3) | 0.158 | 11656(29.4) | 0.053 |
| Heart failure | 1135 (16.9) | 35531 (59.4) | 0.973 | 5503 (24) | 0.178 | 7878(19.9) | 0.078 |
| Pulmonary heart disease | 572 (8.5) | 12652 (21.1) | 0.361 | 2052 (9.0) | 0.016 | 3241(8.2) | 0.012 |
| Cerebrovascular diseases | 543 (8.1) | 10180 (17.0) | 0.272 | 3630 (15.8) | 0.240 | 4004(10.1) | 0.071 |
| *Chronic kidney diseases* |  |  |  |  |  |  |  |
| Stage 3 | 777 (11.5) | 16679 (27.9) | 0.419 | 4440 (19.4) | 0.218 | 5514(13.9) | 0.071 |
| Stage 4 | 139 (2.1) | 3514 (5.9) | 0.196 | 1719 (7.5) | 0.257 | 1406(3.5) | 0.090 |
| End stage renal disease | 159 (2.4) | 926 (1.5) | 0.059 | 1490 (6.5) | 0.202 | 1091(2.8) | 0.025 |
| *Others* |  |  |  |  |  |  |  |
| Obstructive sleep apnea | 2422 (36) | 16696 (27.9) | 0.174 | 3067 (13.4) | 0.543 | 12293(31) | 0.106 |
| Mood disorders | 1981 (29.4) | 15099 (25.2) | 0.095 | 4309 (18.8) | 0.25 | 11981(30.2) | 0.017 |
| Liver cirrhosis | 180 (2.7) | 2492 (4.2) | 0.082 | 735 (3.2) | 0.032 | 1249(3.2) | 0.028 |
| Schizophrenia | 108 (1.6) | 1421 (2.4) | 0.055 | 573 (2.5) | 0.063 | 897(2.3) | 0.048 |
| Lung cancer | 95 (1.4) | 2139 (3.6) | 0.139 | 975 (4.3) | 0.172 | 746(1.9) | 0.037 |
| **Medications (%)** |  |  |  |  |  |  |  |
| Glucocorticoids | 3513 (52.2) | 31406 (52.5) | 0.005 | 10201 (44.5) | 0.154 | 19386(48.9) | 0.066 |
| Biguanides | 1922 (28.6) | 14630 (24.4) | 0.094 | 7330 (32) | 0.074 | 13437(33.9) | 0.116 |
| Insulin | 1764 (26.2) | 28984 (48.4) | 0.472 | 9537 (41.6) | 0.329 | 14588(36.8) | 0.23 |
| Sulfonylureas | 467 (6.9) | 5453 (9.1) | 0.080 | 3501 (15.3) | 0.268 | 4186(10.6) | 0.128 |
| Thiazolidinediones | 109 (1.6) | 885 (1.5) | 0.011 | 757 (3.3) | 0.109 | 791(2.0) | 0.028 |
| Alpha glucosidase inhibitors | 10 (0.1) | 72 (0.1) | 0.008 | 287 (1.3) | 0.133 | 73(0.2) | 0.009 |
| **Laboratory** |  |  |  |  |  |  |  |
| BMI ≥ 25 kg/m2 | 4983 (74.1) | 38429 (64.2) | 0.215 | 10496 (45.8) | 0.603 | 28161(71.1) | 0.067 |
| HbA1c ≥ 9 % | 644 (9.6) | 6471 (10.8) | 0.041 | 2428 (10.6) | 0.034 | 5722(14.4) | 0.150 |

*Values are mean ± SD or n (%). GLP-1RA: Glucagon-like peptide-1 receptor agonists; DPP-4i: Dipeptidyl peptidase-4 inhibitors; COPD: Chronic Obstructive Pulmonary Disease; HbA1c: Glycated Hemoglobin; BMI: body mass index; standardized mean difference (SMD)

**Supplementary Table 7. Absolute differences in 365-day Kaplan–Meier estimated cumulative incidence and descriptive number-needed-to-treat(NNT)–equivalent estimates for tirzepatide versus comparator therapies.**

|  |  | **Absolute difference in 365-day cumulative incidence, percentage points** | **Descriptive NNT–equivalent estimate** |
| --- | --- | --- | --- |
|  | **SGLT2i (N= 6,080)** |  |  |
|  | Primary outcome: COPD exacerbation | 2.80 | 36 |
|  | Secondary outcomes: |  |  |
| All-cause mortality | | 4.06 | 25 |
| Acute respiratory failure | | 8.57 | 12 |
| Lower respiratory tract infection | | 3.56 | 29 |
|  | **DPP-4i (N=4,684)** |  |  |
|  | Primary outcome: COPD exacerbation | 2.88 | 35 |
|  | Secondary outcomes: |  |  |
|  | All-cause mortality | 6.06 | 17 |
|  | Acute respiratory failure | 8.99 | 12 |
|  | Lower respiratory tract infection | 6.57 | 16 |
|  | **GLP-1RA (N=6,727)** |  |  |
|  | Primary outcome: COPD exacerbation | 0.18 | NA^*^ |
|  | Secondary outcomes: |  |  |
|  | All-cause mortality | 1.39 | 72 |
|  | Acute respiratory failure | 0.66 | NA^*^ |
|  | Lower respiratory tract infection | −0.04^+^ | NA^*^ |

Absolute differences were calculated as the Kaplan–Meier estimated cumulative incidence in the comparator cohort minus that in the tirzepatide cohort at day 365 and are expressed in percentage points. Positive values indicate a lower estimated cumulative incidence in the tirzepatide cohort. For statistically significant comparisons with a positive absolute difference, the descriptive NNT-equivalent was calculated as the reciprocal of the absolute difference expressed as a proportion and rounded up to the nearest whole number. Given the observational study design, these estimates are descriptive measures of the observed associations and should not be interpreted as causal treatment effects.

COPD, chronic obstructive pulmonary disease; DPP-4i, dipeptidyl peptidase-4 inhibitor; GLP-1RA, glucagon-like peptide-1 receptor agonist; NA, not applicable; NNT, number needed to treat; SGLT2i, sodium–glucose cotransporter-2 inhibitor; TZP, tirzepatide.

*A descriptive NNT-equivalent estimate was not reported when the corresponding between-group association was not statistically significant.

^+^The negative absolute difference indicates a numerically higher Kaplan–Meier estimated cumulative incidence at day 365 in the tirzepatide cohort; the difference was minimal and not statistically significant.

**Supplementary Table 8. Results for Negative Control Outcomes**

|  | **HR (95%CI)** | **Log-rank test *p*** |
| --- | --- | --- |
| **TZP vs SGLT2i** |  |  |
| Skin cancer | 0.88 (0.62, 1.24) | 0.4562 |
| Cataract | 1.03 (0.78, 1.37) | 0.8191 |
| **TZP vs DPP-4i** |  |  |
| Skin cancer | 0.70 (0.48, 1.02) | 0.0638 |
| Cataract | 1.15 (0.85, 1.59) | 0.3608 |
| **TZP vs GLP-1RA** |  |  |
| Skin cancer | 1.00 (0.71, 1.40) | 0.9887 |
| Cataract | 0.97 (0.75, 1.25) | 0.7952 |
